# Hourly 5-km surface total and diffuse solar radiation in China, 2007–2018

**DOI:** 10.1038/s41597-020-00654-4

**Published:** 2020-09-23

**Authors:** Hou Jiang, Ning Lu, Jun Qin, Ling Yao

**Affiliations:** 1 State Key Laboratory of Resources and Environmental Information System, Institute of Geographic Sciences and Natural Resources Research, Chinese Academy of Sciences, Beijing, 100101 China; 2 College of Resources and Environment, University of Chinese Academy of Sciences, Beijing, 100190 China; 3 Southern Marine Science and Engineering Guangdong Laboratory, Guangzhou, 511458 China; 4 Jiangsu Center for Collaborative Innovation in Geographical Information Resource Development and Application, Nanjing, 210023 China

**Keywords:** Climate change, Atmospheric science

## Abstract

Surface solar radiation is an indispensable parameter for numerical models, and the diffuse component contributes to the carbon uptake in ecosystems. We generated a 12-year (2007–2018) hourly dataset from Multi-functional Transport Satellite (MTSAT) satellite observations, including surface total solar radiation (R_s_) and diffuse radiation (R_dif_), with 5-km spatial resolution through deep learning techniques. The used deep network tacks the integration of spatial pattern and the simulation of complex radiation transfer by combining convolutional neural network and multi-layer perceptron. Validation against ground measurements shows the correlation coefficient, mean bias error and root mean square error are 0.94, 2.48 W/m^2^ and 89.75 W/m^2^ for hourly R_s_ and 0.85, 8.63 W/m^2^ and 66.14 W/m^2^ for hourly R_dif_, respectively. The correlation coefficient of R_s_ and R_dif_ increases to 0.94 (0.96) and 0.89 (0.92) at daily (monthly) scales, respectively. The spatially continuous hourly maps accurately reflect regional differences and restore the diurnal cycles of solar radiation at fine resolution. This dataset can be valuable for studies on regional climate changes, terrestrial ecosystem simulations and photovoltaic applications.

## Abstract (summary)

**Table:** 

Measurement(s)	stellar radiation • global solar radiation • diffuse solar radiation
Technology Type(s)	satellite imaging of a planet • neural network model
Factor Type(s)	year of data collection • hourly, daily and monthly radiation measurements
Sample Characteristic - Environment	climate system
Sample Characteristic - Location	China

Machine-accessible metadata file describing the reported data: 10.6084/m9.figshare.12864251

## Background & Summary

In recent years, research on quantitative estimation of surface total solar radiation (R_s_) and diffuse solar radiation (R_dif_) has attracted growing interest in view of its great scientific value and socioeconomic benefits^1^. R_s_ is a prerequisite for modelling terrestrial ecosystem productivity^2^, and an essential element for estimating heat fluxes, soil moisture and evapotranspiration^3^. The distribution and intensity of R_s_ are required for site selection of solar photovoltaic power and further estimation of power production^4^. Previous studies revealed that R_dif_ contributes to the ecosystem carbon uptake by increasing the canopy light use efficiency^5–7^. The knowledge on R_dif_ is required to assess its impacts on plant productivity and carbon dynamics of terrestrial ecosystems^7–10^. For instance, surface downward direct and diffuse radiation are necessary inputs for Forest Biomass, Assimilation, Allocation, and Respiration (FöBAAR) model to simulate forest carbon cycle^11^. The perturbations of R_dif_ are required when using Yale Interactive terrestrial Biosphere (YIBs) model to study the response of global carbon cycle to fire pollutions^12^. Besides, the fraction of diffuse and direct solar radiation as well as their variations are essential for modelling radiation-use efficiency of wheat during its vegetative phase^13^ and the early assessment of crop (i.e., soybean, wheat and sunflower) yield on a daily or shorter basis^14^.

Although great efforts have been made to establish globally covered surface-radiation networks, such as the Baseline Surface Radiation Network (BSRN), World Radiation Data Centre (WRDC) and Global Energy Balance Archive (GEBA), it remains insufficient to derive high-resolution radiation estimates from measurements alone because of the sparsity and heterogeneity of stations^15^. Since meteorological variables are commonly available and easily accessible, empirical models such as temperature-based, sunshine duration-based, as well as relative humidity- and cloud-based models are developed to extend R_s_ estimates to more meteorological stations^2,16^, but their accuracies are strongly affected by measurements under insufficient calibration schedule^17^. Retrieval from satellite observations is the most reliable way to gain spatially continuous estimates of R_s_ as digital signals on sensors carry massive information about the atmospheric state and underlying land surface^18^. These algorithms include two categories: constructing empirical relationships between top of atmosphere and surface radiative fluxes^19,20^, and driving radiative transfer models by utilizing satellite-derived atmospheric parameters^1,21^.

Several global R_s_ datasets have been generated through satellite retrievals. For instance, the Global LAnd Surface Satellite (GLASS)^22^ provides global 5-km resolution, 3-h interval R_s_; Tang *et al*.^23^ produced a 16-year dataset (2000–2015) of high-resolution (3 h, 10 km) global R_s_. Nevertheless, none of them provide estimate of R_dif_. In addition, large uncertainties frequently occur under broken clouds due to the neglect of adjacency effect in their pixel-based retrieval schemes^24–26^ that depend on an assumption of plane-parallel homogeneous clouds. However, this assumption does not always hold. For example, in the presence of broken clouds, multiple reflections and scattering events off the sides of clouds lead to significant photon transport^27–29^, which makes great difference at fine scales where R_s_ of an individual footprint under inhomogeneous clouds is relevant to multiple adjacent satellite pixels^24^. Therefore, area-to-point retrievals seem the optimal solutions, i.e., adjacent signals within a certain extent are involved for radiation estimation.

The notable progress of deep learning in modelling spatial context opens new perspectives^30^. Convolutional neural networks (CNN) have been widely utilized to extract spatial features from satellite images for definition and classification of extreme situations, for instance, storms, spiral hurricanes, and atmospheric rivers^31^. Thus, it is feasible to capture the spatial distribution of clouds/aerosols through CNNs for handling spatial adjacent effects caused by photon transport. In our previous work, a deep network consisting of CNN module and multi-layer perceptron (MLP) has been developed for R_s_ estimation for the first time^32^, and achieved breakthrough of data accuracy at hourly scale. In this study, we further extend the previous network to fit the requirements of R_dif_ estimation through transfer learning, and then use the newly trained network and previous one to generate high-resolution (hourly, 5 km) R_s_ and R_dif_ time series data in China. The final published dataset^33^ includes R_s_ and R_dif_ at hourly, daily and monthly scales from 2007 to 2018. This unique data source are useful for analysis of regional characteristics and temporal cycles of solar radiation at fine scales, as well as radiation-related applications or scientific researches particularly climate changes and utilization of renewable solar energy.

## Methods

### Basic data

**Fig. 1:**
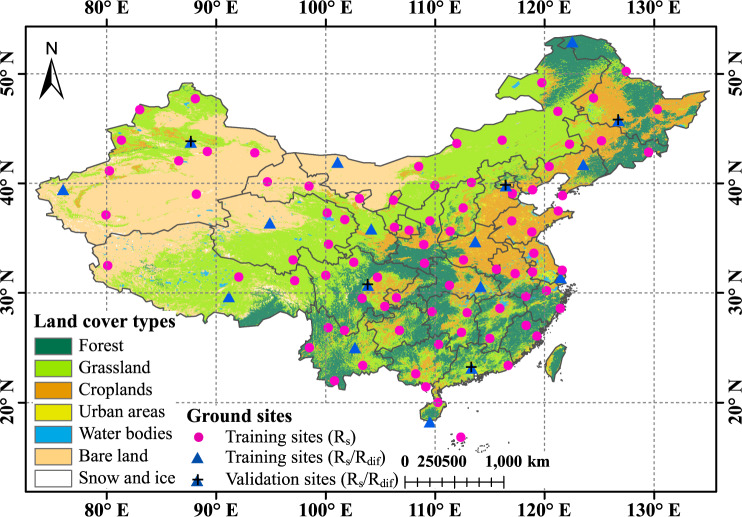
Locations of radiation stations used in our study. Triangles with black cross are used for independent model validation while others are for model training or fine-tuning. The background land cover types are derived from MODIS MCD12Q1 products in 2008.

To train the proposed deep network, training samples should be prepared at first. The output corresponds to ground measurements of R_s_ or R_dif_. The inputs include satellite image blocks and associated attributes of time (month, day, and hour) and location (latitude, longitude and altitude). Hourly R_s_ and R_dif_ measurements are available from China Meteorological Administration (CMA) (http://data.cma.cn/ last accessed: 11 Jan. 2020). The used hourly records involve 98 radiation stations and cover a period from 1 Jan. 2007 to 31 Dec. 2008. The data in 2008 were used for training of deep network while that in 2007 were for independent validation. Figure 1 shows the spatial distribution of all related stations, of which 81 sites (circles) only provide R_s_ while the rest 17 sites (triangles) provide both R_s_ and R_dif_. These stations locate in different climate zones and their background land cover types include forests, grasslands, croplands, bare lands etc., ensuring the representativeness of training samples for deep network. A simple physical threshold test^34^ was adopted to exclude the spurious and erroneous measurements. In total, 0.49% of all records not passing the test were deleted and 441547 samples for R_s_ and 55096 samples for R_dif_ were retained for subsequent experiments. Besides, daily and monthly records of 98 radiation stations from 2007 to 2014 were used for validation of time-series products. Their quality was controlled based on the reconstructed daily and monthly integrated R_s_ data^35^.

The used satellite images are Multi-functional Transport Satellites (MTSAT) data provided by the Japan Meteorological Agency (JMA). The MTSAT-1R, positioned at 104°E above the equator, scans the surface every 30 minutes and provides images over Asia-Pacific region (70°N–20°S, 70°E–160°E) in five channels: one visible channel (VIS, 0.55–0.80μm), two split-window channels (IR1, 10.3–11.3μm; IR2, 11.5–12.5μm), one water vapour channel (IR3, 6.5–7.0μm) and one shortwave infrared channel (IR4, 3.5–4.0μm). The original MTSAT-1R satellite data are resampled to so-called hourly GAME products with a resolution of 0.05°, which is freely accessible at http://weather.is.kochi-u.ac.jp/ (last accessed: 11 Jan. 2020). We utilized the visible channel of GAME products to estimate target radiation, i.e., R_s_ and R_dif_.

Finally, altitude of each pixel should be determined thus DEM data are required. DEM data are from Shuttle Radar Topography Mission that generates the most complete high-resolution digital topographic database of the Earth, covering over 80% of the Earth’s land surface between 60°N and 56°S. The data can be obtained from the website http://srtm.csi.cgiar.org/srtmdata/ (last accessed: 11 Jan. 2020). The original DEM data with data points posted approximately 30 m were resampled to grids with 0.05° resolution. DEM data provide elevation information for gridded inputs during spatially continuous estimation.

### Estimation of surface solar radiation

The method we used to estimate surface solar radiation is mainly based on the CNN-based deep network developed in our previous work^32^. The network is demonstrated to be effective in handling spatial adjacent effects of surface radiation and simulating complicated radiative transfer processes and to be successful in retrieving accurate estimation of R_s_ from geostationary satellite data. In summary, the deep network consists of CNN module and MLP. CNN module takes image blocks as inputs thereby allowing identical treatment of adjacent satellite pixels, and is further stacked to construct deep residual structure to extract hierarchical features from low-level details (e.g., geometric shapes, sizes, orientations, edges and distribution) to high-level comprehensive abstract representations (e.g., intrinsic physical and optical properties of mixed clouds). Such hierarchical architecture of spatial features is a reflection of the scattering and absorption effects as well as their interactions in the atmosphere; hence, it can be considered as substitutes for input parameters in radiative transfer models to describe atmospheric state. The MLP is utilized to link extracted features of CNN and additional auxiliary information (involving the state in time and space) to target hourly R_s_ through implicit non-linear expressions, whose parameters are learnt from pre-prepared training samples in a supervised manner. Traditional physical algorithms retrieve surface radiation from satellite signals through various radiative transfer models or their simplified versions, where geometric/atmospheric conditions and aerosol types should be strictly defined, complex atmospheric processes need to be precisely simulated, and clear-sky and cloudy retrieval modes are independently developed. In contrast, all-sky situations are under a unified framework in our CNN-based algorithm and tedious intermediate simulations are avoided. Another advantage is that the deep network is capable of handling spatial adjacent effects of surface radiation, in other words, considering the influence of neighbouring pixels on radiation estimation of the central point. More details on the network structure and the spatial adjacent effects can refer to refs. ^32,36^.

**Fig. 2:**
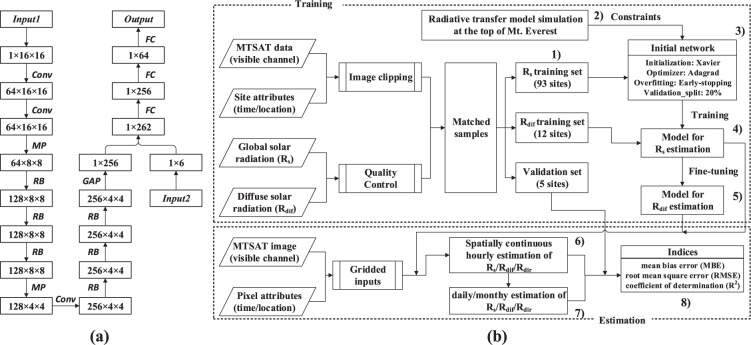
Framework used to generate radiation datasets. (**a**) The structure of our deep network. *Conv* represents convolution operation, *MP* means max-pooling operation, *RB* is the abbreviation of residual blocks, and *GAP* stands for global average-pooling operation. The size of three-dimensional blocks is labelled below as channels × width × height. (**b**) The flowchart to generate datasets and analyse outputs. Numbers 1–8 note the main procedures.

The graphical structure of the proposed deep network is illustrated in Fig. 2a. There are two input flows: *Input1* for satellite image blocks and *Input2* for additional attributes corresponding to the central point of *Input1*. The *Output* is target R_s_ associated with the central point of *Input1*. More details can refer to ref. ^32^. The input size for CNN is 16 × 16 pixels (~80 × 80 km on the ground) based on the recommendation that time series of satellite pixels are most correlative within an extent of approximately 60 km at hourly scale^25^ and our previous experiments on the spatial scale effect of satellite-based R_s_ estimation^36^. This setting also fits in the requirements of classical CNN structure and ensures the extraction of edge features. In addition, only visible band of satellite data is utilized for the convenience of cross-sensor applications because visible channel is available for nearly all satellite sensors. It is reasonable as visible channel provides the most proportion of information on aerosols, clouds and other atmospheric properties^20^.

In our previous experiment, an outstanding deep network for R_s_ estimate has been obtained after continuous trial-and-error process and iterative parameter optimization. Herein, we further fine-tune the previous network for the sake of R_dif_ estimation using new training samples consisting of ground measured R_dif_ and corresponding satellite image block. The transfer learning was adopted to overcome the problem associated with insufficient R_dif_ samples. The parameters for convolutional layers (*Conv*) were initialized from the trained R_s_ model while that for fully-connected layers (*FC*) were reset to zero. Therefore, R_dif_ samples were mainly responsible for MLP fitting. Training and tuning processes were the same as R_s_. In this way, the best model for R_dif_ estimation can be obtained in short time as CNN module has mastered the rules to abstract spatial pattern from satellite image blocks. After model learning and optimization, the trained R_dif_ model in combination with previous R_s_ model was used to generate our radiation datasets.

### Workflow of data generation

The schematic flowchart to generate our radiation datasets is illustrated in Fig. 2b. The entire workflow consists of two main sections: training and estimation. The codes and datasets for training and estimation process can be accessed at the figshare^37^ (10.6084/m9.figshare.c.4891302). The training section concentrates on learning the underlying non-linear relationships between satellite images and measured surface radiation, and outputs two deep networks for R_s_ and R_dif_ estimation. The estimation section predicts spatially continuous R_s_ and R_dif_ data using the trained networks by feeding gridding inputs. The main procedures are numbered in Fig. 2b and described as follows:

1. Prepare training sets. For each ground station, a 16 × 16 neighbouring block was cut out from GAME image and matched up with quality-controlled R_s_ and R_dif_ record in 2008 according to time attributes. These samples were separated into three groups: R_s_ training set (93 training sites in Fig. 1), R_dif_ training set (12 triangle training sites in Fig. 1) and validation set (5 triangle validation sites with black cross in Fig. 1).
2. Simulate the state at the top of Mt. Everest. To guarantee a reasonable extrapolation of the deep network at high altitudes, constraints from radiative transfer model simulation at the top of Mt. Everest were mixed into the R_s_ and R_dif_ training set. The Santa Barbara DISORT Atmospheric Radiative Transfer (SBDART) model was adopted for the simulation^20^.
3. Initialize the deep network. The network was implemented using *keras* package^38^. All parameters of the network were initialized through Xavier^39,8^. The learning rate was initially 0.01 but multiplied by 0.5 across a learning plateau.
4. Train deep network for R_s_ estimation. The Adagrad optimizer^40^ was used to iteratively find the optimal weights and biases that minimize the mean-squared error between the network’s predictions and the training targets. An early-stopping mechanism was utilized to relieve overfitting by relinquishing further optimization when the performance ceased to improve sufficiently. During training process, 20% of the paired samples were randomly selected to serve as a validation set to identify whether the network was overfitting. The model with the best performance was preserved for subsequent estimates.
5. Fine-tune the preserved model in 4) for R_dif_ estimation. Similarly, the model with the best performance was preserved.
  More parameter configurations of step 2–5 can refer to ref. ^32^.
6. Generate spatially continuous hourly estimation. Hourly gridded GAME products from 2007 to 2018 were associated with corresponding time/location attributes, and then the best models in 4) and 5) were used to simultaneously obtain R_s_ and R_dif_ maps by feeding gridded inputs. In addition, surface direct solar radiation (R_dir_) was derived by subtracting R_dif_ from R_s_.
7. Integrate daily and monthly estimates. The missing hourly value was filled by multiplying the corresponding hourly extraterrestrial radiation by the averaged clearness index calculated from available hourly estimates within the day. After that, daily values were sums of all hourly estimates within the day, and monthly values were the sum of all daily values within the corresponding month.
8. Validate radiation datasets. The spatial extensibility of deep network was evaluated using the validation set in 1) that was not involved at training phase. The accuracy of our datasets was further evaluated at hourly scale by comparing to ground measurements in 2007. Moreover, daily and monthly estimates were evaluated using station records from 2007 to 2014. Three indices were used to quantify data quality: correlation coefficient (R), mean bias error (MBE), and root-mean-squared error (RMSE) between estimates and ground measurements:

$${\rm{R}}=\frac{\mathop{\sum }\limits_{i=0}^{n}({y}_{i}-\bar{y})({y}_{i}^{{\rm{{\prime} }}}-\bar{y\text{'}})}{\sqrt{\mathop{\sum }\limits_{i=0}^{n}{({y}_{i}-\bar{{\rm{y}}})}^{2}}\sqrt{\mathop{\sum }\limits_{i=0}^{n}{({y}_{i}^{{\rm{{\prime} }}}-\bar{y\text{'}})}^{2}}}$$

$${\rm{MBE}}=\frac{1}{n}\mathop{\sum }\limits_{i=0}^{n}({y}_{i}^{{\prime} }-{y}_{i})$$

$${\rm{RMSE}}=\sqrt{\frac{1}{n}\mathop{\sum }\limits_{i=0}^{n}{({y}_{i}^{{\prime} }-{y}_{i})}^{2}}$$

where *n* is the total number of data samples indexed by *i*, *y* represents the measured value whose mean value is $\bar{y}$, and $y{\prime}$ is the predicted value with mean $\bar{y{\prime} }$. Relative values of MBE and RMSE (rMBE and rRMSE) were also used.

### Sensitivity analysis

The crucial step of this algorithm is to equip the deep network with the ability to extract abstract spatial pattern from satellite images. The representativeness and balance of training samples and the input size of satellite image blocks affect the reliability of gained pattern for R_s_ estimation, thus the accuracy of estimated data. The 98 stations under different climates and with diverse land cover types guarantee the representativeness of R_s_ training samples. To overcome the imbalance of samples, image blocks corresponding to high radiation values whose proportion is usually small were first rotated by 90/180/270 degrees and flipped up and down, left and right, then several copies of these samples were mixed into the full training set. The investigation of spatial scale effects in ref. ^36^ suggests an optimal input size of 16 × 16 pixels.

Configurations of hyper-parameters were referenced to classical classification and object detection networks in computer vision, for example, the rectified linear unit (ReLU) was used as the activation function as it is effective in alleviating vanishing gradient problems and speeding up learning process; the early-stopping was adopted to prevent overfitting thus it was not necessary to control training epochs carefully. Other sensitive hyper-parameters (listed in Table 1) were determined based on a hierarchical search. To reduce the computational cost associated with the learning procedure of deep network, our experiments were conducted using a small training dataset (twelve training sites with blue triangles in Fig. 1). We first investigated different choices of the learning rate with a fixed configuration for other parameters (the first choice in the search space). After the optimal choice of learning rate (Initial value of 0.01 and multiplied by 0.5 after 10 epochs’ plateau of validation loss) was determined, we continued searching for the optimizer, then the dropout rate and batch size. For learning rate, optimizer and dropout rate, the choice (the bold one in the search space) with the best validation accuracy at the five independent stations in terms of R and RMSE was finally selected. With respect to the batch size, it seems that the smaller size, the better performance but the longer time. Therefore, we chose the intermediate size of 500 for a balance between the performance and time consumption.

**Table 1:** Search for the optimal hyper-parameters of deep network.

Parameter	Search space	R	RMSE (MJ/m^2^)	Time (minute)
Learning rate	Constant value of 0.01	0.858	0.486	
	**Initialized as 0.01 and multiplied by 0.5 after 10 epochs’ plateau of validation loss**	**0.879**	**0.447**	
	Initialized as 0.01 and multiplied by 0.2 after 10 epochs’ plateau of validation loss	0.876	0.449	
	Initialized as 0.01 and multiplied by 0.1 after 10 epochs’ plateau of validation loss	0.510	0.871	
Optimizer	Mini-batch gradient descent (SGD)	0.879	0.447	
	**Adaptive gradient (Adagrad)**	**0.886**	**0.419**	
	Adaptive moment estimation (Adam)	0.807	0.548	
Dropout rate	**0.3**	**0.886**	**0.419**	
	0.1	0.881	0.420	
	0.5	0.885	0.419	
Batch size	**500**	0.886	0.419	25.3
	200	**0.897**	**0.398**	31.3
	1000	0.862	0.460	**20.1**

All experiments are conducted under the framework of *keras*^38^.

## Data Records

All hourly, daily and monthly radiation datasets from 2007 to 2018 are freely available from the Pangaea^33^ at 10.1594/PANGAEA.904136, through which users can link to the specific data entities of each year. The dataset for one year includes twelve folders for hourly radiation (twelve months), one folder for daily total radiation, one folder for monthly total radiation as well as other supporting documents:

- Hourly radiation: twelve zipped folders named as “China_HourlyRadiation _yyyymm.h5”. The hourly files are named as “RAD_yyyymmddhh.h5” and stored as int16 data type in HDF5 format in the unit of 10^−4^ MJ m^−2^. “yyyy”, “mm”, “dd”, and “hh” denote year, month, day and hour (UTC time). Each file contains two variables representing R_s_ and R_dif_, namely global radiation and diffuse radiation, respectively. The time coverage of hourly dataset is from 2007-01-01 0:00 to 2018-12-31 23:00 (UTC).
- Daily and monthly radiation: Daily files are named as “RAD_yyyymmdd.h5” and monthly files are named as “RAD_yyyymm.h5” where “yyyy”, “mm”, and “dd” denote year, month, and day. Values are stored as floating-point data type in the unit of 10^−2^ MJ m^−2^. Each file contains two variables representing R_s_ and R_dif_, namely daily/monthly total global radiation and daily/monthly total diffuse radiation, respectively.

The datasets provide gridded radiation estimates within 71°E–141°E and 15°N–60°N with an increment of 0.05° (about 5 km). The hourly radiation can also be expressed in unit of W/m^2^ through the conversion: 0.01 MJ m^−2^ hour^-1^ = 1/0.36 W m^−2^. More details and examples of data visualization can refer to the published description files in each dataset. It is stressed that all hourly data are provided in UTC time.

## Technical Validation

### Spatial mapping

**Fig. 3:**
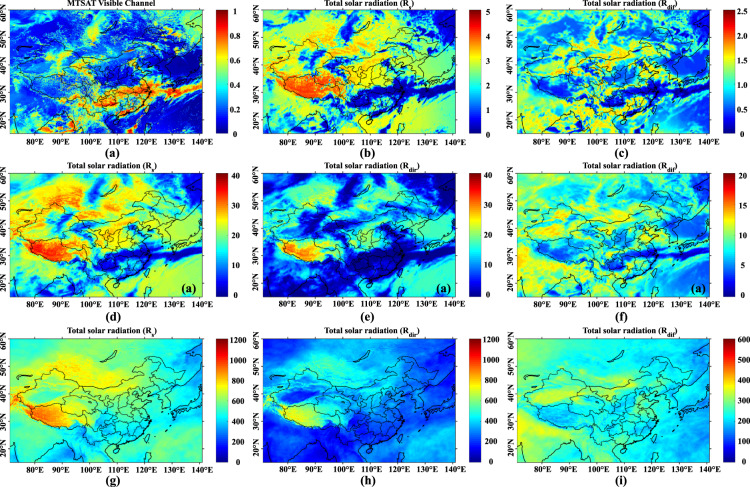
An example from our dataset. (**a**–**c**) Reflectance of the visible channel, hourly R_s_ and hourly R_dif_ at UTC 6:00, 22 Jun. 2018 (BJT 14:00, 22 Jun. 2018). (**d**–**f**) Daily R_s_, R_dir_ and R_dif_ on 22 Jun. 2018. (**g**–**i**) Monthly R_s_, R_dir_ and R_dif_ in June 2018. The unit of radiation is MJ m^−2^.

Figure 3a shows the instantaneous atmospheric state in visible channel captured by MTSAT at UTC 6:00, 22 Jun. 2008 (BJT 14:00, 22 Jun. 2008). The estimated hourly R_s_ and R_dif_ are displayed in Fig. 3b,c, respectively. The influence of cloud depth, surface topography and elevation are reflected in the spatial distribution of surface radiation. Under the thick clouds (red regions in Fig. 3a), both R_s_ and R_dif_ are lower than surrounding areas. In contrast, with respect to regions below thin clouds (yellow regions in Fig. 3a), R_s_ is relatively higher as more R_dif_ is obtained on the surface. For areas under clear sky conditions (blue regions in Fig. 3a), R_s_ is larger in high altitude areas (e.g., the Tibetan Plateau). Figure 3d–i illustrates the spatial distribution of R_s_, R_dir_ and R_dif_ at daily and monthly scales. Daily radiation on 22 June 2008 shares similar characteristics with hourly radiation, indicating a stable atmospheric state in the day. At monthly scale, regional differences are revealed thoroughly. The distribution of solar radiation exhibits obvious latitudinal dependency, but also affected by the surface topography, regional climate and distance to coastal line. In June, R_s_ is highest on the Tibetan Plateau and lowest in the Szechwan and south China due to the significant difference of R_dir_. Conversely, R_dif_ has the minimum value on the Tibetan Plateau while the maximum value locates on the North China Plain. R_dir_ is predominant in regions with high altitudes (the Tibetan Plateau) or drought climate zones (the Mongolia Plateau) while R_dif_ occupies the main proportion for areas with abundant rainfall or frequent cloud coverage (the middle and lower reaches of the Yangtze River, the Szechwan Basin and Guizhou). Although deep networks used for estimation are trained by samples within China, they also provide reasonable estimation in surrounding areas. For example, in June R_dif_ contributes to the majority of surface radiation in India and Southeast Asia due to the coming rainy season.

### Temporal variations

**Fig. 4:**
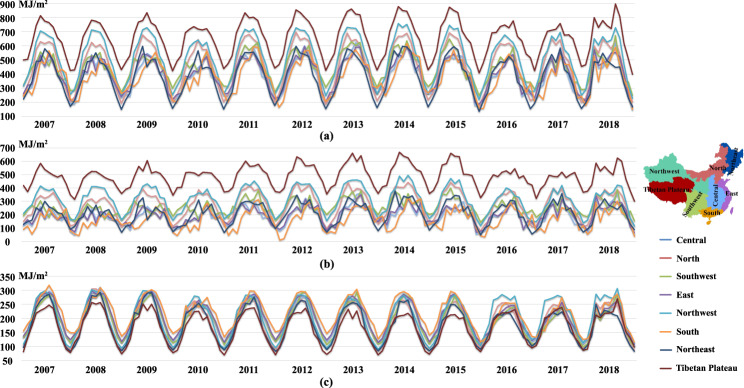
Monthly variations in different regions from 2007 to 2018. (**a**) R_s_, (**b**) R_dir_ and (**c**) R_dif_. The values are from the monthly products. Different regions are as shown on the right.

We establish time series products to observe the temporal variations of surface solar radiation. Figure 4 shows the monthly variations of statistically averaged R_s_, R_dir_ and R_dif_ for different regions in China from 2007 to 2018. R_s_ on the Qinghai-Tibet Plateau is the highest all the year round, benefiting from significantly higher altitudes, which in contrast leads to the lowest received R_dif_ as shown in Fig. 4c. The proportion of R_dif_ exhibits the highest in the south of China (relatively lower R_s_ but higher R_dif_) compared with other regions due to the frequent cloudy and rainy weather. A slight dimming of R_s_ is observed in 2010, followed by the brightening from 2011 to 2015, and then by a dimming from 2016 to 2017. Howbeit the long-term trends of R_dif_ are inconsistent with the variations of R_s_. For instance, neither obvious brightening nor dimming is manifested in the northwest while a decreasing tendency continues until 2015 on the Qinghai-Tibet Plateau. The fluctuation of R_dir_ is more obvious than R_dif_, accounting for the overall variations of R_s_, because both absorption and scattering of the atmosphere lead to decrease of R_dir_ while changes of R_dif_ radiation result from scattering of the atmosphere alone.

### Validation against ground measurements

**Fig. 5:**
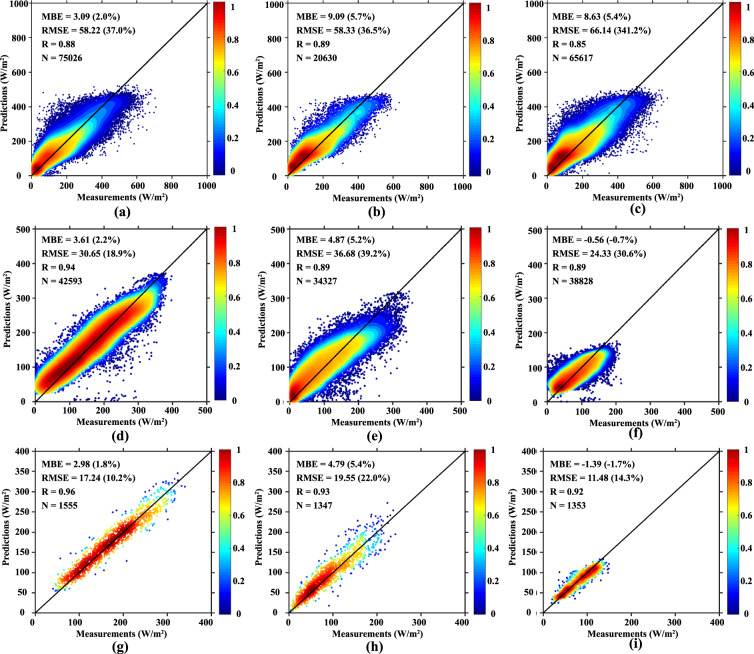
Density plots of our estimates versus measured values. (**a**–**c**) Evaluation of R_dif_ at 12 training sites, at five independent validation sites in 2008 and at all 17 sites in 2007, respectively; (**d**–**f**) daily mean results of R_s_, R_dir_ and R_dif_ from 2007 to 2014; (**g**–**i**) monthly mean results of R_s_, R_dir_ and R_dif_ from 2007 to 2014. The black solid lines are 1:1 lines. The values in brackets indicate rMBE or rRMSE. Gaussian kernels are used for density plots and density values are normalized to 0–1 through min-max normalization.

The validation in our previous work^32^ has demonstrated the outstanding performance of the hybrid deep network on estimation of R_s_. Herein, we evaluate the model performance for R_dif_ estimation to check the viability of transfer learning. The evaluation process includes three stages: performance over training samples (12 triangle training sites in Fig. 1), independent spatial extensibility in 2008 (5 triangle validation sites with black cross in Fig. 1), and temporal extensibility in 2007 at all 17 stations, as shown in Fig. 5a–c. Overall, it provides good estimates for R_dif_ at the site scale with an R of 0.88, MBE of 3.09 W/m^2^ and RMSE of 58.22 W/m^2^ over training samples. The results with an R of 0.89, MBE of 9.09 W/m^2^ and RMSE of 58.33 W/m^2^ at five independent validation sites, and an R of 0.85, MBE of 8.63 W/m^2^ and RMSE of 66.14 W/m^2^ in 2007, are comparable to the training phase, revealing the powerful spatial and temporal extensibility of deep networks in estimating R_dif_. The positive MBE values confirm that our datasets overestimate R_dif_ at some degree, which might attribute to relative lower measured values due to instrument drifting sensitivity and urbanization effects^41,42^. In fact, it is a challenging task to estimate R_dif_ due to much higher demands for fully consideration of aerosols, clouds, and their interactions. Yet for all that, our estimates of R_dif_ (Fig. 5c) outperform the widely-used ERA5 reanalysis data released by European Centre for Medium-Range Weather Forecasts (ECMWF) which has an R of 0.85, negative MBE of 43.08 W/m^2^ and RMSE of 96.93 W/m^2^ when evaluated at the same CMA diffuse radiation stations in 2007^42^.

Furthermore, our datasets are evaluated against ground measurements collected at 98 CMA radiation stations from 2007 to 2014 at daily mean and monthly mean scales as shown in Fig. 5d–i. Our daily results of R_s_ at the spatial resolution of 5 km exhibit an R of 0.94, MBE of 3.61 W/m^2^ and 30.65 W/m^2^. The intrinsic difference between point nature of ground measurements and areal average of gridded radiation products usually takes part of the responsibility for above deviations^24^. At a finer spatial resolution of 5 km the RMSE of our daily R_s_ is still superior to widely-used products such as the ISCCP-FD data at 2.5° resolution with an R of 0.89 and RMSE of 68.3 W/m^2^ (see Section 3.1 of ref. ^36^), the GEWEX-SRB data at 1° spatial resolution with an R of 0.91 and RMSE of 36.5 W/m^2^ (see Section 4 of ref. ^18^), and recent ISCCP-HXG products at 10 km resolution with an R of 0.93 and RMSE of 32.4 W/m^2^ (see Table 3 of ref. ^23^) which were also validated against observations at the CMA radiation stations. At monthly scale, the R value increases to 0.96, 0.93 and 0.92 meanwhile RMSE decreases to 17.24, 19.55 and 11.48 W/m^2^ for R_s_, R_dir_ and R_dif_, respectively, which is also remarkably better than other products (compare to Table 2 of ref. ^36^). It should be pointed out that the excellent performance at monthly scale benefits from the mutual offset of underestimation and overestimation, for instance, daily R_dif_ shows an overestimation in the low-value part and an underestimation in the high-value part (Fig. 5f) while this does not occur for monthly R_dif_ (Fig. 5i).

**Table 2:** Comparative experiments to check potential sampling errors.

No.	Description	R	RMSE (MJ/m^2^)
E1	Training the network using the fully R_s_ training dataset	**0.933**	**0.325**
E2	Training the network using R_s_ measurements at the twelve R_dif_ training sites	0.886	0.419
E3	Training the network using R_s_ measurements at randomly selected twelve training sites	0.717	0.655
E4	Training the network using R_dif_ measurements at the twelve R_dif_ training sites	0.796	0.323
E5	Fine-tuning the trained network of E1 using R_dif_ measurements at the twelve R_dif_ training sites	**0.850**	**0.238**
E6	Fine-tuning the trained network of E1 using K-fold cross-validation strategy. The 17 R_dif_ sites were divided into 4 groups (4-4-4-5), 3 out of which were used to train the network with the rest excluded. The training was repeated four times for all the combinations, and then all simulations of the sites excluded in the four repeats were put together to calculate R and RMSE to measure the performance.	0.712	0.260
E7	A stress test where we used for validation only the five sites that are more humid or with higher elevation, or closer to cities. Other sites were used to fine-tune the trained network of E1.	0.759, 0.584, 0.790	0.333, 0.451, 0.317

The three values for E7 represent results of the three extreme experiments on humidity, elevation and distance to cities, respectively.

### Uncertainties

**Fig. 6:**
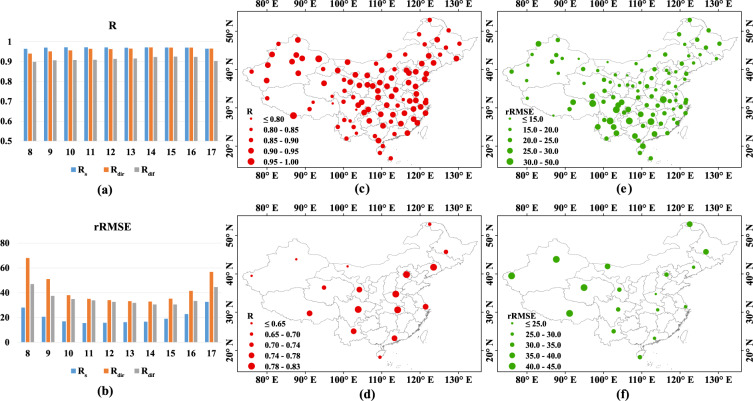
Uncertainties of hourly estimates in time and space. (**a**,**b**) The coefficient *R* and rRMSE in 2007 for different hours from 8:00 to 17:00; (**c**,**d**) Spatial distribution of the coefficient *R* for R_s_ and R_dif_, respectively; (**e**,**f**) Spatial distribution of rRMSE for R_s_ and R_dif_, respectively.

Figure 6a,b shows the errors of hourly estimates grouped by local hours from 8:00 to 17:00. All groups correlate well with the ground measurements with the lowest R being 0.96, 0.93 and 0.87 for R_s_, R_dir_ and R_dif_, respectively, proving the good performance of deep network in hourly radiation estimation. Large rRMSEs are likely to appear in the morning and at night when the amounts of received surface radiation are very low. The data accuracy is acceptable with the average rRMSE lower than 20% (R_s_) or 40% (R_dir_ and R_dif_). It points out that temporal deviations might result from the fact that satellite images reflect an instantaneous state of the atmosphere whereas ground measurements represent an average state within per unit time (herein one hour). When clouds move rapidly, ground stations are likely to be covered by cloud shadows during a momentary period (less than one hour) but satellite sensor may scan a clear sky because clouds have drifted across. In this case, ground measurements would be smaller than satellite-based estimates. Therefore, large positive deviations usually occur when coming across changeable clouds. A limitation of our method is that it is unable to simulate dramatic changes in short time because our trained network just takes into consideration the spatial adjacent effects of solar radiation but ignores the lag effect and cumulative effect in time series. The recurrent neural networks^43,44^ that are able to model temporal dynamic behaviour are the promising solutions.

The R and rRMSE of hourly R_s_ and R_dif_ at each site are displayed in Fig. 6c–f, where obvious geographical differentiation is observed. Overall, our estimates correlate well with ground measurements at sites with high probability of cloud-free skies, for instance, the north and northwest China. Low R and large rRMSE are likely to occur at sites located in regions with cloudy days, such as the south and southwest China, especially the Szechwan Basin perennially covered by clouds. It is known that both dust aerosol particles in the north and northeast China and dense clouds in the south and southeast China lead to non-clear skies, but model performance is inconsistent in these areas. This phenomenon indicates that our developed deep network does well in simulating radiative effects of aerosols, but slightly bad in handling clouds. Such shortcoming is attributed to the inadequate information on cloud properties^45^. As only visible channel of MTSAT satellite is used, it is difficult for deep network to determine optical properties of clouds, thus their interactions with radiation. With the aid of additional channels, better retrieval under cloudy conditions might be possible, for instance, depending on the identification of ice clouds and liquid water clouds whose radiative effects are significantly different. Moreover, the gridded products from satellite data are inherently spatial domain-averaged while ground measurements focus on solar radiation in local areas. This discrepancy always leads to evaluation deviations in space^46^. When ground station is covered by shadows of cirrus clouds, the measured surface solar radiation would be lower than satellite-derived values because the footprint of satellite grid has larger spatial coverage.

With regard to R_dif_, the correlation between our estimates and ground measurements is worse than that of R_s_. Different from R_s_, estimates of R_dif_ behave well in humid areas (southern China) rather than arid areas (northwest China), against our common sense that cloudy weather conditions in the southern China strongly affect the accuracy of radiation estimation. On the premise that deep network for R_s_ estimation has proved its effectiveness in arid areas, the worse performance on R_dif_ estimation under the same framework might be attributed to the poor data quality. Evidence comes from the fact that measurements of R_dif_ in the western China are not in a full-automatic tracking manner but manual operations, of which the nonstandard ones often lead to measurement errors. This contradictory phenomenon also indicates that a small proportion of problematic ground measurements would not affect the performance of deep network owing to its powerful robustness.

### Sampling errors

The representativeness of R_dif_ training samples is worthy of special concern as only measurements at twelve stations are involved. To reduce the influence of insufficient samples on estimated data accuracy, we adopted the transferring learning approach to reuse the rules on how CNN extracts spatial pattern from satellite blocks that have mastered during R_s_ estimation based on a larger dataset. We designed 7 experiments (listed in Table 2) to have an in-depth inspection of potential sampling errors associated with this approach. E1 trains the deep network using the fully R_s_ training dataset. E2 trains the network using R_s_ measurements at the twelve R_dif_ training sites. E3 trains the network using R_s_ measurements at randomly selected twelve training sites. E4 trains the network using R_dif_ measurements at the twelve R_dif_ training sites. E5 fine-tunes the trained network of E1 using R_dif_ measurements at the twelve R_dif_ training sites. The performance of the gained network in E1-E5 is validated at the same five independent sites in terms of R and RMSE on R_s_ or R_dif_. E6 fine-tunes the trained network of E1 through K-fold cross-validation strategy, i.e., the 17 R_dif_ sites were divided into 4 groups (4-4-4-5), and then 3 out of the 4 groups were used to train the network while the rest one was excluded. The training process was repeated four times for all the combinations and the R and RMSE of all predictions of the sites excluded in the four repeats were calculated to measure the performance of E6. E7 is a stress test where we used for validation only the five sites that are more humid or with higher elevation or closer to cities.

The results show that selecting densely and evenly distributed sites is the only way to improve the generalization ability of deep network (cf. E1 and E2), but it is also beneficial to make the limited sites distributed in representative areas with diverse characteristics (cf. E2 and E3, E5 and E6). Although the comparison is conducted on R_s_, we assume it bears valid information for R_dif_ as well. Regardless of the small number, diffuse radiation stations cover all typical climate zones in China (Fig. 1), maximizing their spatial representation as much as possible; hence, it is rational to believe in the reliability of the trained network for R_dif_ estimation. Compared with training a network for R_dif_ estimation from the beginning (E4), fine-tuning the trained R_s_ network through transferring learning (E5) makes up the limitation caused by insufficient R_dif_ samples to a certain extent. Anyhow, the comparison between E1 and E6 demonstrates the existence of sampling errors and suggests that R_dif_ estimation requires further attempts and efforts. The stress test (E7) gave us an idea of the maximum sampling error. Since R_dif_ is highly influenced by humidity (function of climate and vegetation) and probably pollution and altitude, we pertinently removed sites that are more humid or with higher elevation, or closer to cities from training samples, but used them only for validation. Due to the inevitable reduction of the representativeness of training samples, the validation accuracy was lower than that of E5. These extreme cases show that the expected maximum sampling error of our R_dif_ estimates may not exceed the worst value of E7, i.e., R of 0.584 and RMSE of 0.451 MJ/m^2^. Anyhow, such sampling errors announce the importance to collect more representative R_dif_ measurements for improving the performance of deep network on R_dif_ estimates.

## Usage Notes

Datasets can be reused as stand-alone for analysis of regional characteristics and temporal trend of solar radiation, yet richer studies and applications can be done by linking to other data resources. A simple direction is comparing this dataset to other products (e.g., ERA5^42^, BESS^26^, GLASS^22^ etc.) to account for merits and demerits of different approaches for radiation estimation, or gain new understanding in typical regions (e.g., the Tibetan Plateau). We also suggest the open-source Global Solar Energy Estimator (GSEE) model^47^ (www.github.com/renewablesninja/gsee) for accurate estimation of solar energy in China to help policy-making of energy sector^48^. If data on residential rooftop locations, electricity consumption and price, capital investment etc. are available, a comprehensive assessment of resource, technical, economic and market potential of rooftop solar photovoltaics^49^ can be conducted based on our high-resolution (5 km) radiation dataset. Besides, there exists the possibility to drive plant models (e.g., JULES^7^, YIB^50^, SWAP^51^ etc.) for crop yield estimation^13^.

## Data Availability

The MATLAB codes for spatial visualization of files in HDF format are published along with our datasets in PANGAEA. The codes and datasets for training and estimation process can be accessed at the figshare^37^ (10.6084/m9.figshare.c.4891302).
